# Supplementary material for: Short-term associations of diarrhoeal diseases in children with temperature and precipitation in seven low- and middle-income countries from Sub-Saharan Africa and South Asia in the Global Enteric Multicenter Study
Source: PLoS Negl Trop Dis. 2024 Oct 15;18(10):e0011834. doi: 10.1371/journal.pntd.0011834 (PMC11510124; doi:10.1371/journal.pntd.0011834)
Supplement: S1 Table — (DOCX) [file pntd.0011834.s001.docx]

**S1 Table. Summary statistics information of ERA5 land**

| Country | Time zone | Temperature (°C) | |  | Precipitation (mm) | | |  |
| --- | --- | --- | --- | --- | --- | --- | --- | --- |
|  |  | Median | IQR |  | Median | IQR | Number of days with 0 mm rain (%) |  |
| The Gambia | UTC±00.00 | 28.2 | 4.4 |  | 0.0 | 3.9 | 727 (64.9) |  |
| Mali | UTC±00.00 | 26.7 | 4.6 |  | 0.3 | 7.7 | 575 (52.3) |  |
| Mozambique | UTC+02.00 | 23.7 | 5.0 |  | 1.2 | 3.9 | 396 (35.3) |  |
| Kenya | UTC+03.00 | 22.0 | 1.3 |  | 9.7 | 14.3 | 73 (6.4) |  |
| India | UTC+05.30 | 27.4 | 5.4 |  | 1.0 | 16.4 | 537 (48.9) |  |
| Bangladesh | UTC+06.00 | 26.8 | 5.7 |  | 3.1 | 21.2 | 467 (42.3) |  |
| Pakistan | UTC+05.00 | 27.6 | 6.2 |  | 0.0 | 0.2 | 891 (81) |  |

IQR: Interquartile range
