# Supplementary material for: Short-term associations of diarrhoeal diseases in children with temperature and precipitation in seven low- and middle-income countries from Sub-Saharan Africa and South Asia in the Global Enteric Multicenter Study
Source: PLoS Negl Trop Dis. 2024 Oct 15;18(10):e0011834. doi: 10.1371/journal.pntd.0011834 (PMC11510124; doi:10.1371/journal.pntd.0011834)
Supplement: S2 Table — (DOCX) [file pntd.0011834.s002.docx]

**S2 Table. Summary of age-specific relative risk of temperature (°C) and precipitation over 21 days on all-cause diarrhoea for seven countries at 95^th^ percentile temperature and precipitation.**

| Country | Temperature RR | | |  | Precipitation RR | | |
| --- | --- | --- | --- | --- | --- | --- | --- |
|  | 0-11  months | 12-23  months | 24-59  months |  | 0-11  months | 12-23  months | 24-59  month |
| The Gambia | 1.36 | 2.23 | 0.59 |  | 1.14 | **1.73*** | 1.29 |
| Mali | **0.18*** | 0.51 | 0.47 |  | 1.07 | **2.41*** | **2.48*** |
| Mozambique | **3.30*** | **8.98*** | 2.31 |  | 0.99 | 0.85 | 0.69 |
| Kenya | 2.13 | 5.39 | 7.99 |  | 1.53 | 1.01 | 2.07 |
| India | 0.76 | 0.79 | 3.07 |  | 1.01 | **1.74*** | **2.96*** |
| Bangladesh | **14.98*** | **6.82*** | 4.85 |  | 1.23 | 1.33 | 0.79 |
| Pakistan | **0.27*** | **0.09*** | **0.25*** |  | **0.76*** | 0.86 | 0.87 |

Temperature-Reference at 1st percentile temperature, Precipitation- Reference at 1st percentile Precipitation, *significance at 5% level.
