# Supplementary material for: Short-term associations of diarrhoeal diseases in children with temperature and precipitation in seven low- and middle-income countries from Sub-Saharan Africa and South Asia in the Global Enteric Multicenter Study
Source: PLoS Negl Trop Dis. 2024 Oct 15;18(10):e0011834. doi: 10.1371/journal.pntd.0011834 (PMC11510124; doi:10.1371/journal.pntd.0011834)
Supplement: S3 Table — (DOCX) [file pntd.0011834.s003.docx]

S3 Table. List of site-specific meta predictors

| Country | ¥Temperature | ¥Precipitation | ¥GDP  Per Capita | ¥Population  Density | ¥Poverty | ¥Urbanization | Treated  Water  (%) | Drinking  Water  (%) | Handwash  (%) | ORS  Used  (%) | *Stunting | *Virus | *Bacteria | *Protozoa | *MSD |
| --- | --- | --- | --- | --- | --- | --- | --- | --- | --- | --- | --- | --- | --- | --- | --- |
| Gambia | 28.8  (2.8) | 5.48  (15.8) | 1993  (270) | 52  (35) | 89.29  (6.12) | 1.001153  (0.017) | 35.86 | 19.73 | 73.86 | 7.09 | 26.04 | 42.27 | 59.38 | 26.82 | 26.78 |
| Mali | 27.5  (3.12) | 6.9  (13.7) | 1654  (39) | 177  (1129) | 91.4  (15.56) | 1.395949  (0.426) | 0.79 | 0.1 | 78.41 | 10.77 | 18.4 | 22.53 | 41.17 | 37.73 | 39.09 |
| Mozambique | 23.5  (3.23) | 4.89  (11.8) | 1524  (789) | 143  (747) | 86.55  (13.7) | 1.008403  (0.091) | 9.1 | 0.44 | 68.43 | 26.73 | 31.86 | 38.33 | 55.36 | 40.53 | 10.82 |
| Kenya | 22  (0.96) | 13.4  (13.6) | 1530  (176) | 258  (365) | 81.06  (9.44) | 1.000904  (0.026) | 62.2 | 4.61 | 94.17 | 13.69 | 28.73 | 28.79 | 57.25 | 28.73 | 87 |
| India | 26.4  (3.97) | 11.4  (20.7) | 3664  (200) | 2836  (5849) | 53.18  (15.54) | 1.21645  (0.4) | 43.49 | 0.77 | 51.02 | 37.31 | 26.66 | 44.71 | 46.17 | 31.82 | 48.03 |
| Bangladesh | 25.5  (3.78) | 15.6  (27.8) | 2189  (590) | 1354  (646) | 72.21  (8.95) | 1.07783  (0.072) | 4.38 | 99.5 | 87.66 | 56.67 | 24.03 | 27.91 | 82.14 | 18.94 | 23.25 |
| Pakistan | 26.2  (3.84) | 1.5  (8.63) | 5082  (0) | 1305  (5564) | 78.04  (15.7) | 1.16812  (0.338) | 46.98 | 0 | 71.46 | 51.43 | 47.38 | 42.37 | 70.59 | 28.46 | 7.49 |

¥ mean and SD (standard deviation); *prevalence rate (PR); Oral Rehydration Solution (ORS); MSD: moderate-to-severe diarrhoea.

We included site-specific indicators related to socioeconomic factors and public health. GDP per capita is sourced from Gridded Gross Domestic Product (per capita) data from [Dryad](https://datadryad.org/stash/dataset/doi:10.5061/dryad.dk1j0). Population density is sourced from [Gridded Population of the World](https://sedac.ciesin.columbia.edu/data/set/gpw-v4-population-density-adjusted-to-2015-unwpp-country-totals-rev11) (GPW) using UN WPP Adjusted Population Density. Poverty levels are derived from the [Global Gridded Relative Deprivation Index](https://sedac.ciesin.columbia.edu/data/set/povmap-grdi-v1/data-download) (GRDI) and Urbanization-the gridded global urban land expansion product at a 1-km resolution are derived from [PANGAEA](https://doi.pangaea.de/10.1594/PANGAEA.905890). Other health-related indicators such as treated water, the primary source of drinking water, handwashing practices, the prevalence of stunting, viruses, bacteria, protozoa, moderate to severe diarrhoea (MSD) among under-five children, and percentage of treated Oral Rehydration Solutions (ORS) are sourced from the GEMS case-control study [1].

**Definition of variables and data source:**

Temperature: Mean temperature (°C) during the study period *(Data source-ERA5 Land)* [2]

Precipitation: Daily total Precipitation (mm) *(Data source-ERA5 Land)* [2]

GDP per capita (Mean): Gridded Gross domestic product (per capita) *(Data source -Dryad)* [3]

Population density (Mean): Gridded Population of the World (GPW), number of persons per square kilometer *(Data source -UN WPP Adjusted Population Density, v4.11)* [4]

Poverty (Mean): Gridded Poverty Mapping-level of deprivation. This mapping characterizes the relative levels of multidimensional deprivation and poverty in each 30 arc-second (~1 km) pixel, where a value of 100 represents the highest level of deprivation and a value of 0 the lowest. *(Data source -Global Gridded Relative Deprivation Index (GRDI), v1)* [5]

Urbanization (Mean score): Gridded global urban land expansion product at 1-km resolution for 2015. (Data source: PANGAEA-a Data Publisher for Earth & Environmental Science) [6]

Treated water: Percentage of households used treated water. *(Data source -GEMS case-control study)* [1].

Drinking water: Tube wells are the primary source of drinking water (percentage of households) [1].

Handwash: Percentage of households washing hands with a shop [1].

Stunting: Prevalence of stunting of under-five child *(Data source -GEMS case-control study)* [1]

Virus: Prevalence of virus under-five child *(Data source -GEMS case-control study)* [1]

Bacteria: Prevalence of Bacteria under- five child *(Data source -GEMS case-control study)* [1]

Protozoa: Prevalence of Protozoa under-five child *(Data source -GEMS case-control study)* [1]

MSD: Prevalence of under-five child with moderate to severe diarrhoea *(Data source -GEMS case-control study)* [1]

ORS: Percentage of treated Oral Rehydration Solutions *(Data source -GEMS case-control study)* [1]

**References**

1. Kotloff KL, Nataro JP, Blackwelder WC, Nasrin D, Farag TH, Panchalingam S, et al. Burden and aetiology of diarrhoeal disease in infants and young children in developing countries (the Global Enteric Multicenter Study, GEMS): A prospective, case-control study. The Lancet. 2013;382: 209–222. doi:10.1016/S0140-6736(13)60844-2

2. Muñoz Sabater J. ERA5-Land hourly data from 1950 to present. In: Copernicus Climate Change Service (C3S) Climate Data Store (CDS) [Internet]. 2019 [cited 7 Sep 2023]. doi:10.24381/cds.e2161bac

3. Kummu M, Taka M, Guillaume JHA. Gridded global datasets for Gross Domestic Product and Human Development Index over 1990-2015. Sci Data. 2018;5. doi:10.1038/SDATA.2018.4

4. Center for International Earth Science Information Network - CIESIN CU. Gridded Population of the World, Version 4 (GPWv4): Population Density Adjusted to Match 2015 Revision UN WPP Country Totals, Revision 11. Palisades, New York: NASA Socioeconomic Data and Applications Center (SEDAC); 2018. Available: https://doi.org/10.7927/H4F47M65

5. Center for International Earth Science Information Network - CIESIN CU. Global Gridded Relative Deprivation Index (GRDI), Version 1. Palisades, New York: NASA Socioeconomic Data and Applications Center (SEDAC). Palisades, New York: NASA Socioeconomic Data and Applications Center (SEDAC); 2018. Available: https://doi.org/10.7927/H4F47M65

6. Chen G, Li X, Liu X, Chen Y, Liang X, Leng J, et al. Global projections of future urban land expansion under shared socioeconomic pathways. Nat Commun. 2020;11. doi:10.1038/S41467-020-14386-X
