# Supplementary material for: Short-term associations of diarrhoeal diseases in children with temperature and precipitation in seven low- and middle-income countries from Sub-Saharan Africa and South Asia in the Global Enteric Multicenter Study
Source: PLoS Negl Trop Dis. 2024 Oct 15;18(10):e0011834. doi: 10.1371/journal.pntd.0011834 (PMC11510124; doi:10.1371/journal.pntd.0011834)

**S1 Fig. Time-series plots of the daily number of all-cause diarrhoea cases, moderate to severe diarrhoea cases, daily mean temperature, and precipitation between 2008 and 2011 in the study countries.**


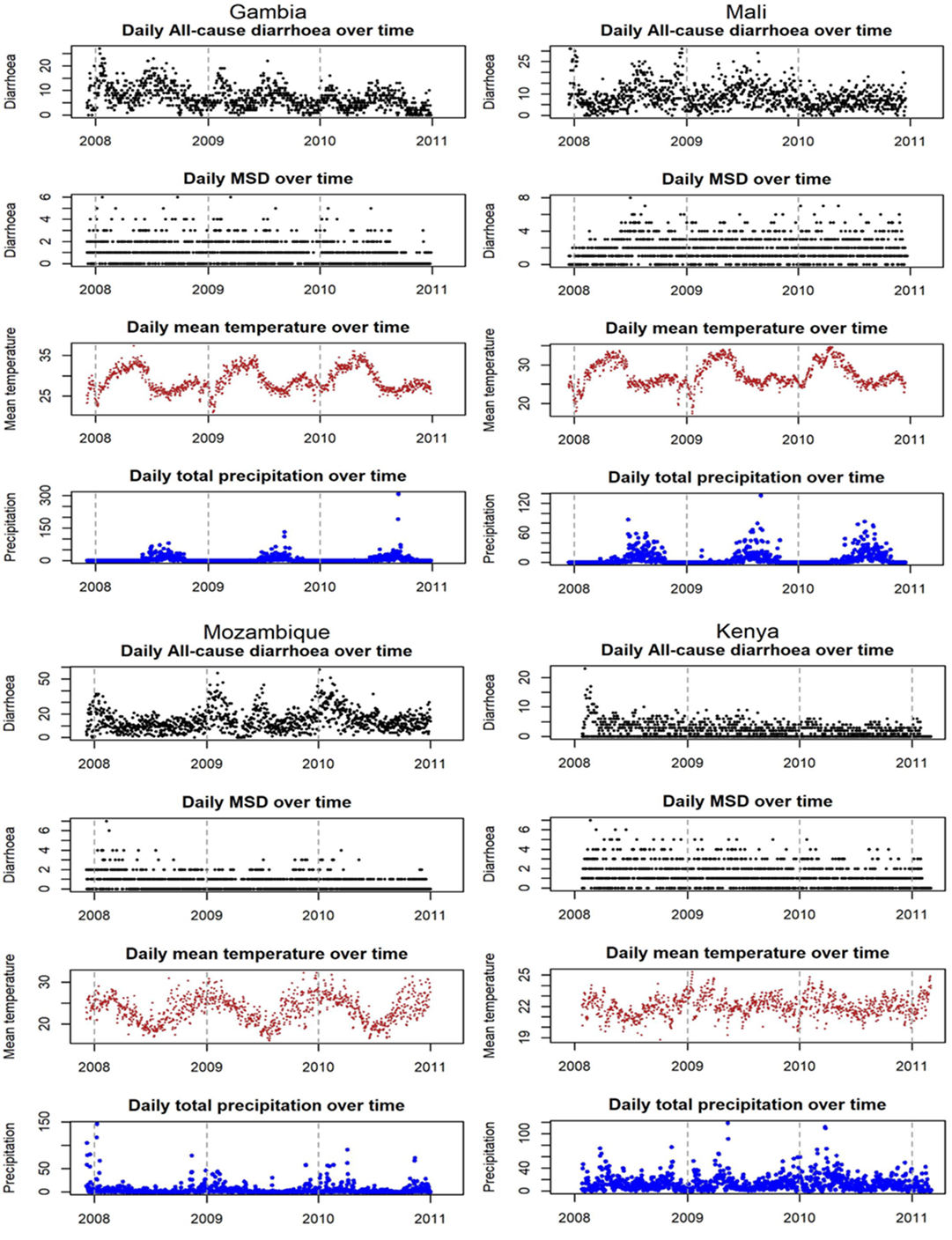


**S1 Fig. (Continue) Time-series plots of the daily number of all-cause diarrhoea cases, moderate to severe diarrhoea cases, daily mean temperature, and precipitation between 2008 and 2011 in the study countries.**


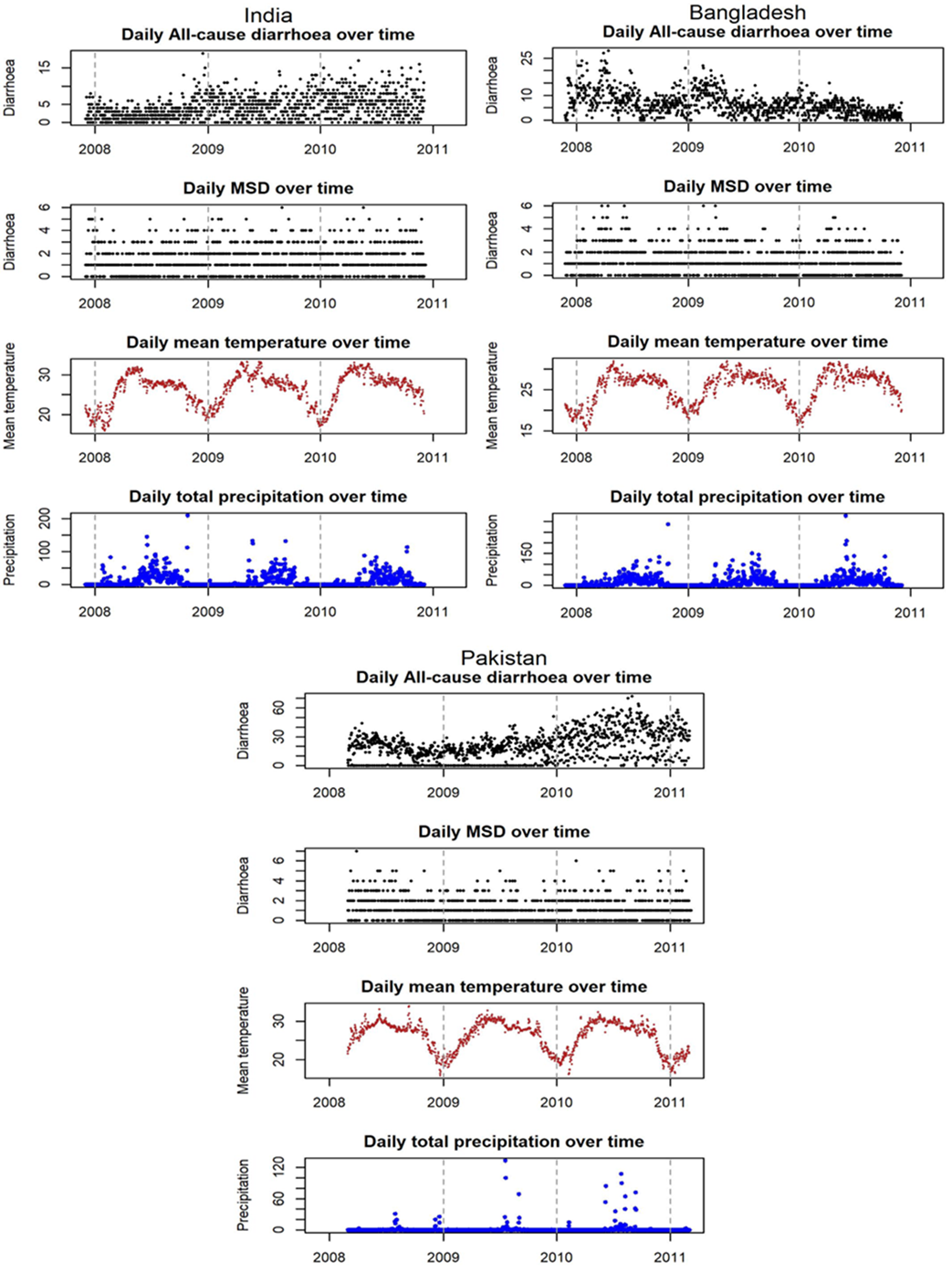

Supplement: S1 Fig — (DOCX) [file pntd.0011834.s004.docx]
