# Supplementary figures and images for: Short-term associations of diarrhoeal diseases in children with temperature and precipitation in seven low- and middle-income countries from Sub-Saharan Africa and South Asia in the Global Enteric Multicenter Study
Source: PLoS Negl Trop Dis. 2024 Oct 15;18(10):e0011834. doi: 10.1371/journal.pntd.0011834 (PMC11510124; doi:10.1371/journal.pntd.0011834)

**S2 Fig. Distribution of ERA5 daily temperature and precipitation.**


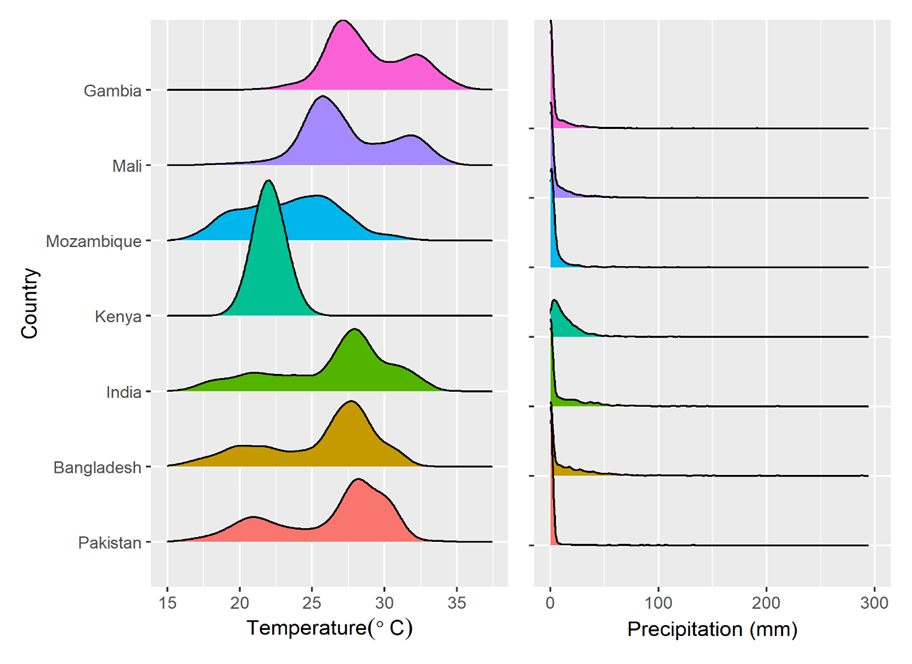

Supplement: S2 Fig — (DOCX) [file pntd.0011834.s005.docx]
