# Supplementary material for: Short-term associations of diarrhoeal diseases in children with temperature and precipitation in seven low- and middle-income countries from Sub-Saharan Africa and South Asia in the Global Enteric Multicenter Study
Source: PLoS Negl Trop Dis. 2024 Oct 15;18(10):e0011834. doi: 10.1371/journal.pntd.0011834 (PMC11510124; doi:10.1371/journal.pntd.0011834)

**S3A Fig. Lag-response relationship between temperature and ACD morbidity at 95^th^ percentile temperature for each site.**


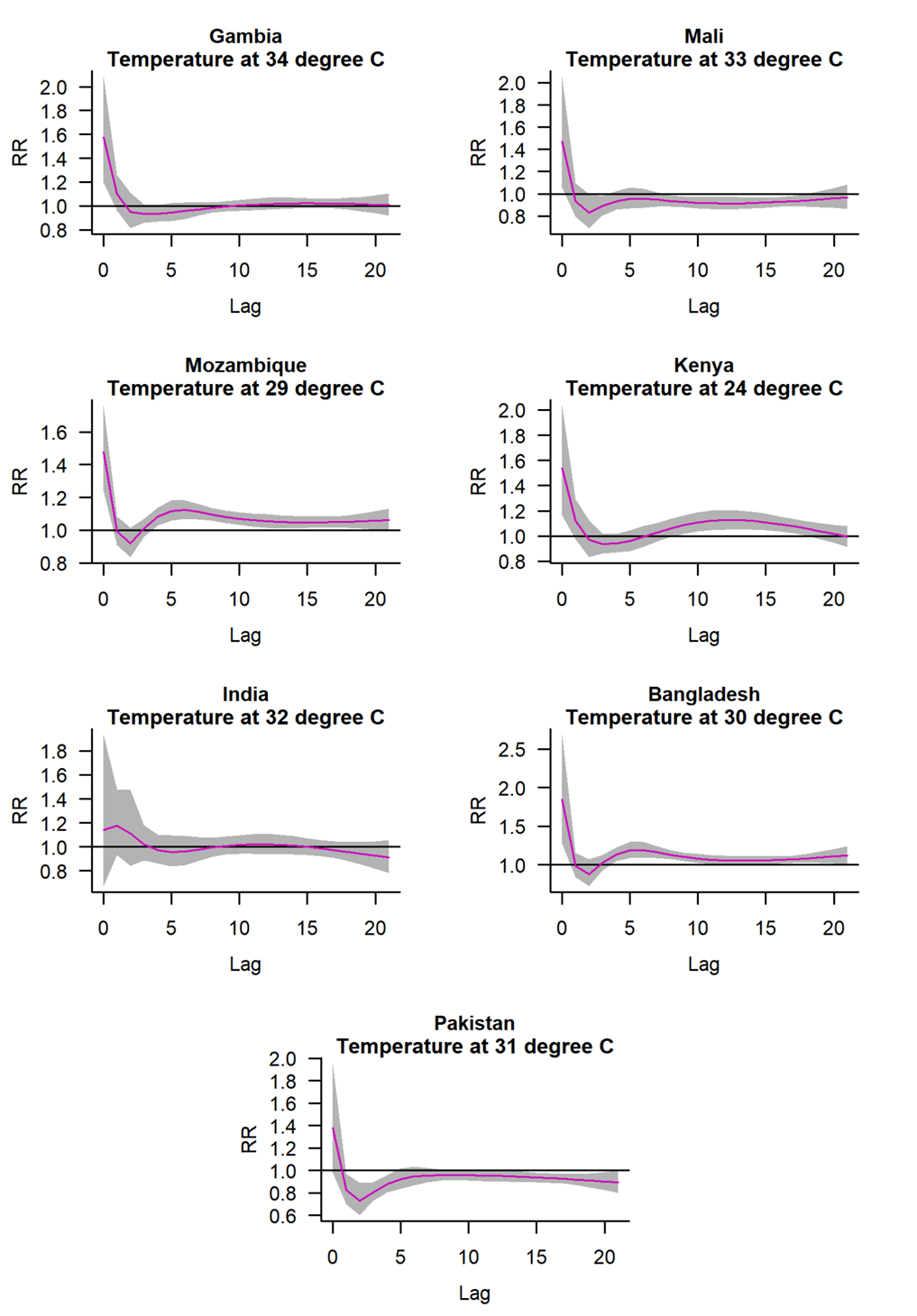


**S3B Fig. Relationships between temperature and ACD specific to lag 7 for each site.**


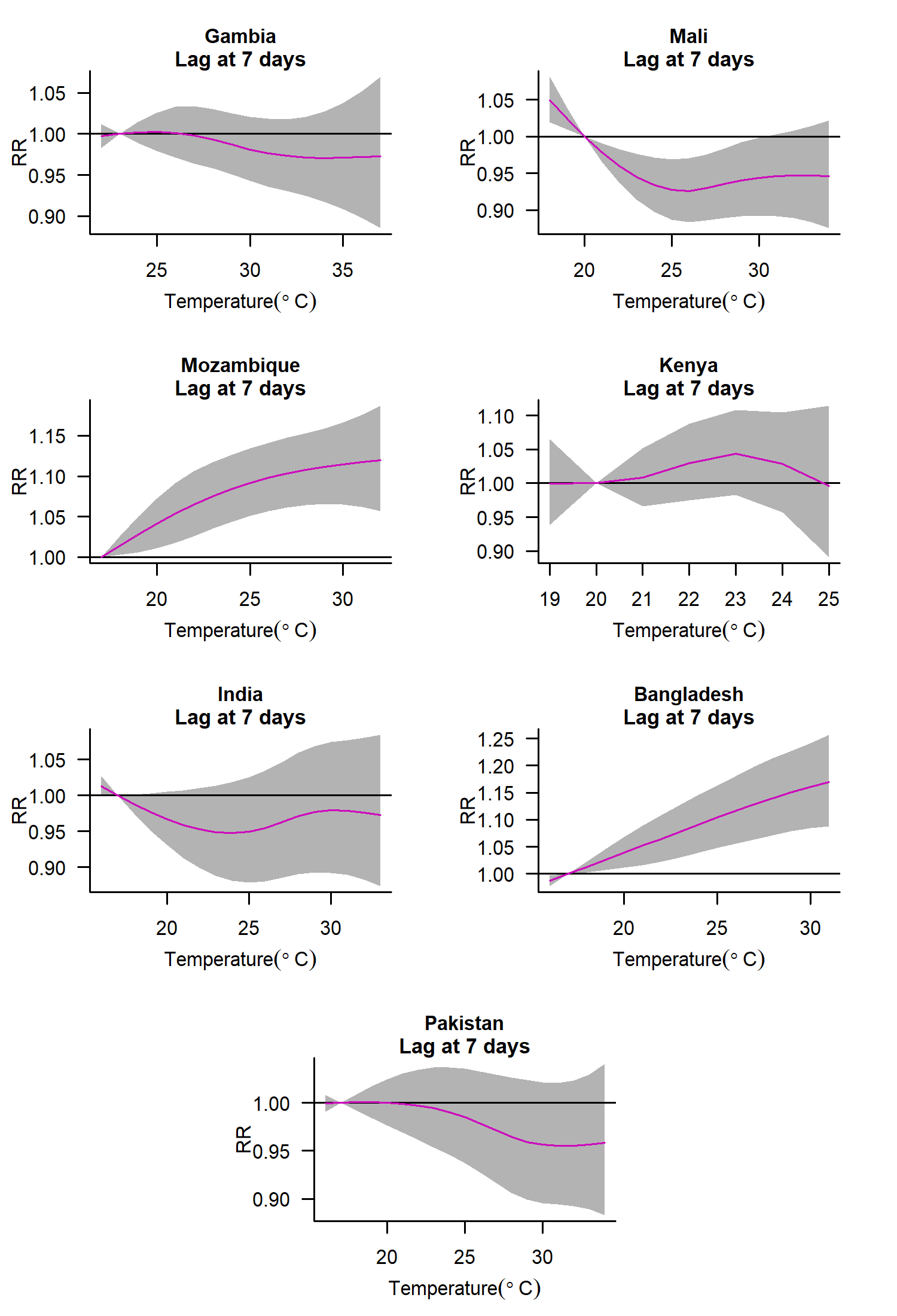


**S3C Fig. Relationships between temperature and ACD specific to lag 14 for each site.**


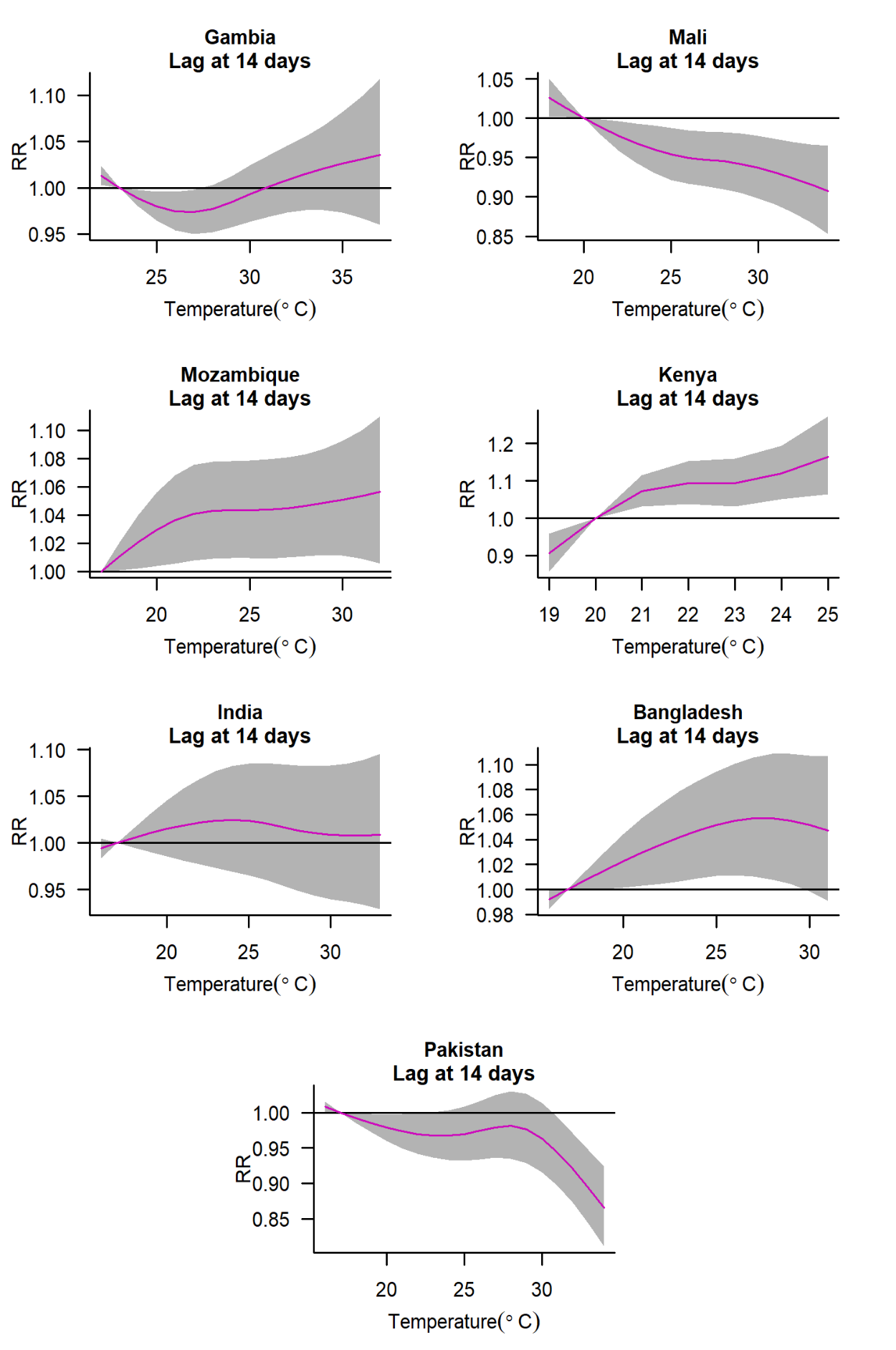


**S3D Fig. Relationships between temperature and ACD specific to lag 21 for each site.**


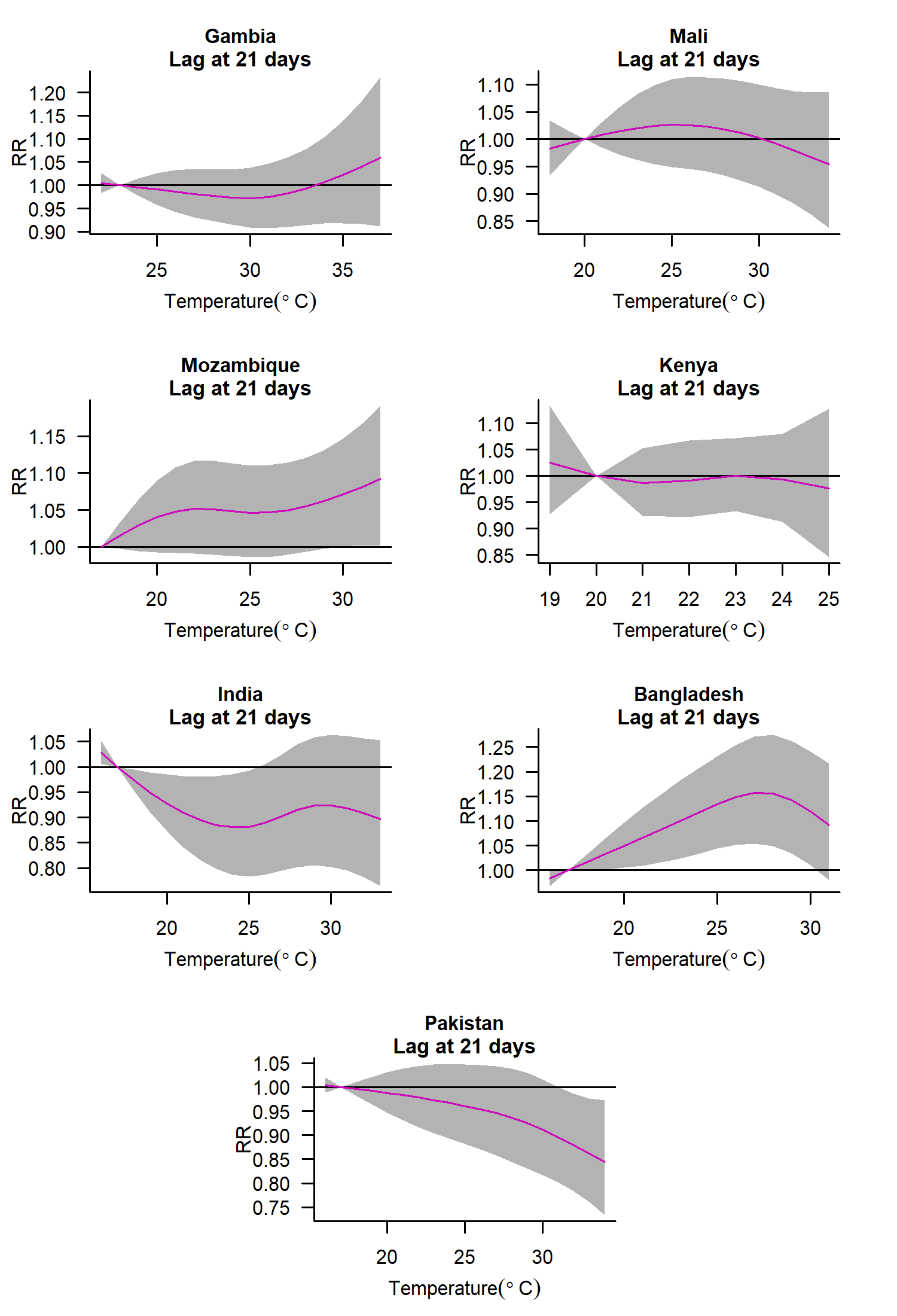

Supplement: S3 Fig — (DOCX) [file pntd.0011834.s006.docx]
