# Supplementary material for: Short-term associations of diarrhoeal diseases in children with temperature and precipitation in seven low- and middle-income countries from Sub-Saharan Africa and South Asia in the Global Enteric Multicenter Study
Source: PLoS Negl Trop Dis. 2024 Oct 15;18(10):e0011834. doi: 10.1371/journal.pntd.0011834 (PMC11510124; doi:10.1371/journal.pntd.0011834)

**S4 Fig. Association between daily mean temperature over 21 days and all-cause diarrhoea by age groups.**

Purple solid pink lines represent point estimates of relative risk (RR) for all ages; the blue dashed line is for 0-11 months age child; the green dashed line is for 12-23 months age child, the yellow dashed line is for 24-59 months age child, the dashed black vertical lines are reference temperature at the 1^st^ percentile and the dashed purple vertical lines are temperature at 95^th^ percentiles.


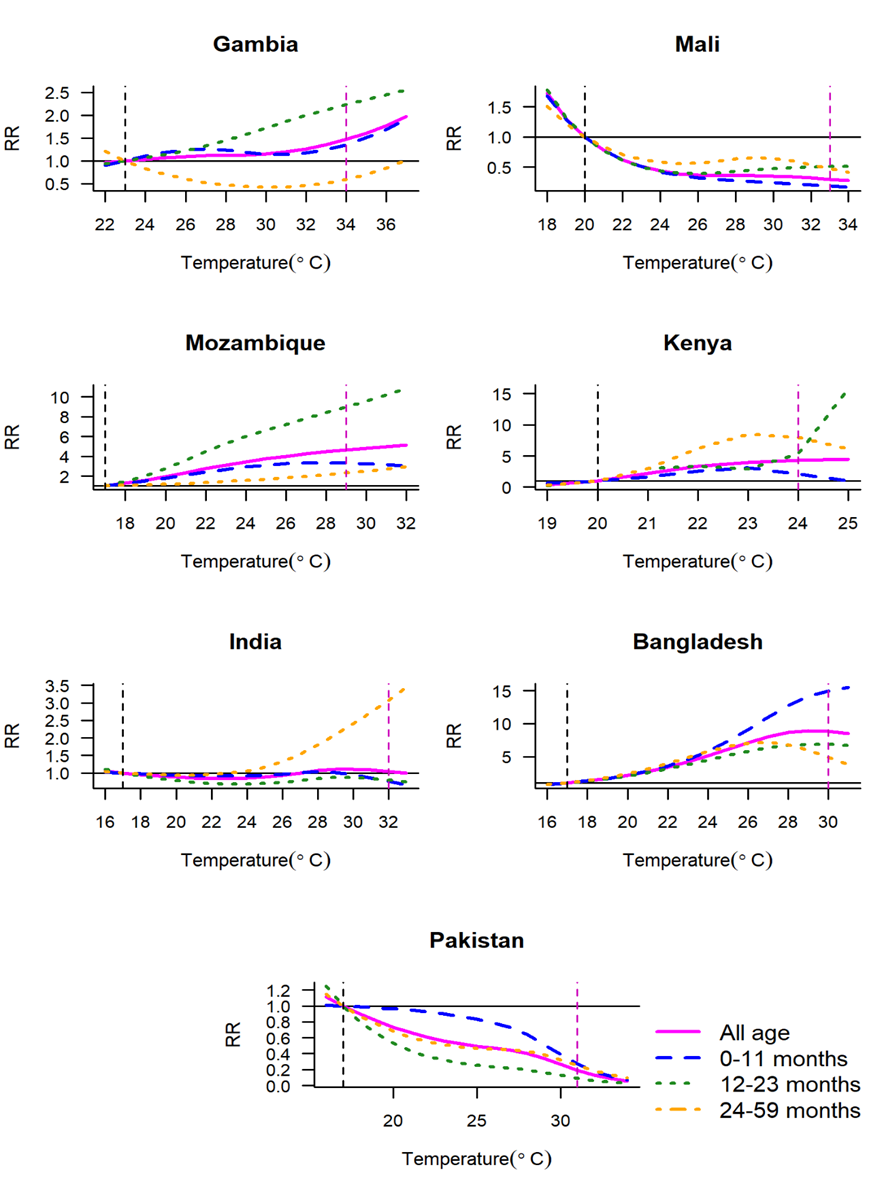

Supplement: S4 Fig — (DOCX) [file pntd.0011834.s007.docx]
