# Supplementary material for: Short-term associations of diarrhoeal diseases in children with temperature and precipitation in seven low- and middle-income countries from Sub-Saharan Africa and South Asia in the Global Enteric Multicenter Study
Source: PLoS Negl Trop Dis. 2024 Oct 15;18(10):e0011834. doi: 10.1371/journal.pntd.0011834 (PMC11510124; doi:10.1371/journal.pntd.0011834)

**S7 Fig. Cumulative relative risks (RRs) with 95% Cis [95^th^ vs. 1^st^] by country in sensitivity analyses for the Precipitation-Diarrhoea model.**


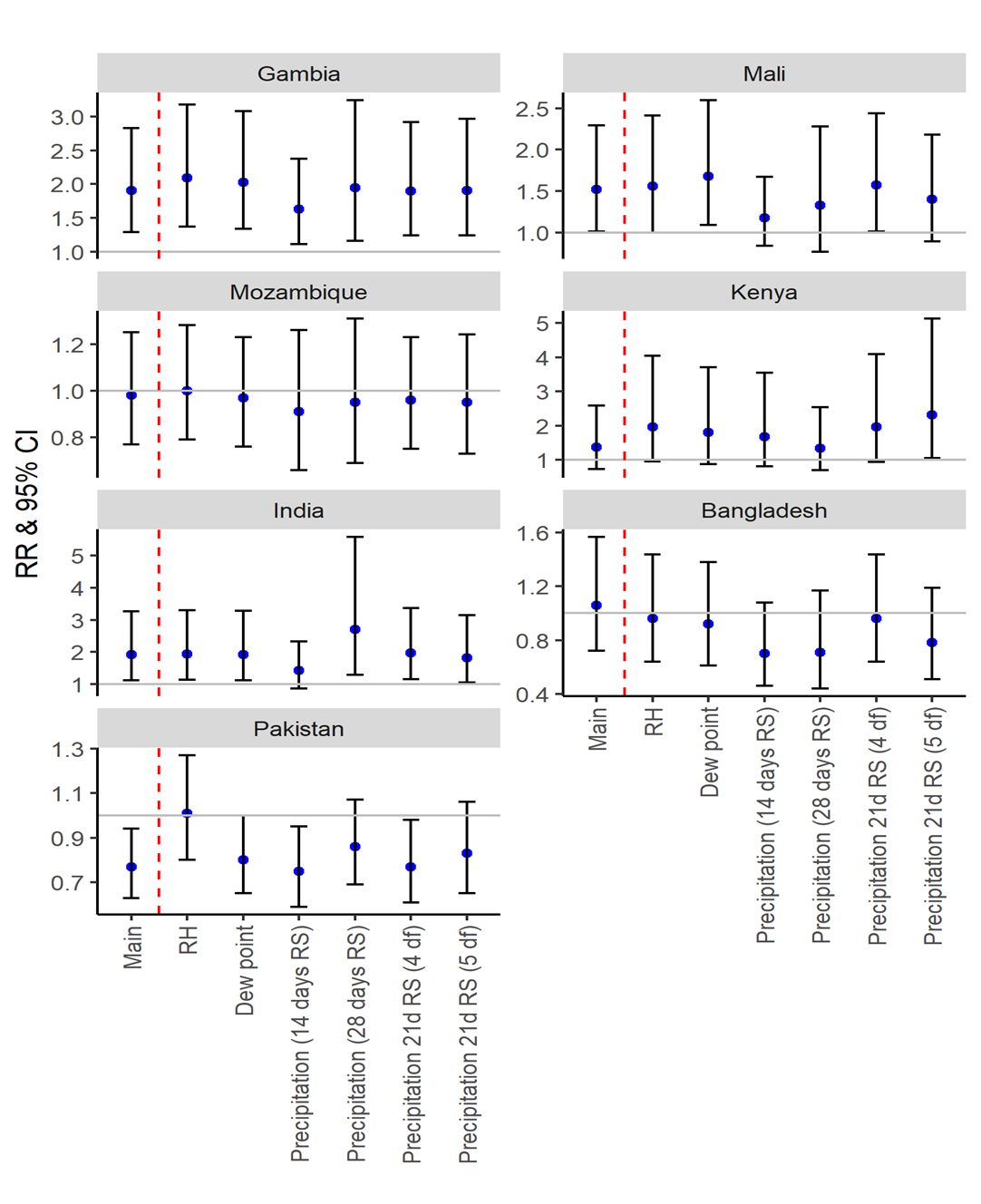

Supplement: S7 Fig — (DOCX) [file pntd.0011834.s010.docx]
