# Supplementary material for: Short-term associations of diarrhoeal diseases in children with temperature and precipitation in seven low- and middle-income countries from Sub-Saharan Africa and South Asia in the Global Enteric Multicenter Study
Source: PLoS Negl Trop Dis. 2024 Oct 15;18(10):e0011834. doi: 10.1371/journal.pntd.0011834 (PMC11510124; doi:10.1371/journal.pntd.0011834)
Supplement: Attachment — Submitted filename: Response_PNTD-D-23-01533.docx [file pntd.0011834.s011.docx]

**Reviewers’ Comments**

**(Manuscript ID:** PNTD-D-23-01533**)**

**Manuscript Title:** Short-term associations of diarrhoeal diseases in children with temperature and precipitation in seven low- and middle-income countries from Sub-Saharan Africa and South Asia in the Global Enteric Multicenter Study

Dear Editor,

Thank you for the opportunity to revise our manuscript and we gratefully acknowledge the reviewers for their insightful comments that undoubtedly would improve the quality of our manuscript. We have now revised the manuscript accordingly. Our point-by-point response to the reviewers’ comments is enclosed below in blue font. The page number refers to the revised version with track changes.

***Reviewer #1’s Comments***

***Methods***

1. The statistical analysis section can be improved by providing a general explanation of how the cross-basis function of temperature and its lags work since this approach for dealing with nonlinearity and lag is quite complex. A citation for this approach might be useful for those interested in looking further.

Response: Thank you so much for your kind suggestions. We have cited Antonio Gasparrini's 2011 paper and shortly explained the cross-basis function of temperature. (**Page 6, Line 154-156**).

2. This is a small number of sites for a meta regression, especially with many covariates. Some discussion of this could be helpful. Based on the current explanation in methods, I'm having trouble understanding the columns in Table 3 of results and the explanation that certain covariates were important predictors for modifying either temperature-diarrhoea or precipiation diarrhoea association. Were the additional variables only included as interactions with temp and precip or am I misunderstanding the meta-regression approach? Please elaborate in methods by explaining what hypotheses are being tested with both the LR test and Q test so this is clear.

Response: Thank you so much for your kind suggestions. We have followed your suggestions and added the explanation for the LR test and Q test. First, we obtained the effect estimates for each site using quasi-Poisson regression models. We found that the associations of diarrhoea with temperature and precipitation varied by site. To identify the possible factors explaining the heterogeneity among the effect estimates, we further used a meta-regression model with RR estimates as the outcome variable and several meta-predictors on different indicators as independent variables in the model. (**Page 7, Line 187-191**).

***Results***

3. I hope my comments about the methods can help clear up how to interpret Table 3.

Response: Once again thank you so much for your valuable feedback. We have addressed them in the method section.

***Conclusions***

4. The conclusions are presented well.

Response: Thank you so much for your kind appreciation words and encouraging notes.

***Editorial and Data Presentation Modifications***

5. (No Response)

**Summary and General Comments**

6. This is a well thought out analysis of both nonlinear and lagged effects of temperature and precipitation on daily all-cause diarrhea using GEMS data. There have been studies that look at nonlinear relationships between temperature and precipitation, their lags, and various diarrheal outcomes, though perhaps as you say, not independent impact of temperature and precipitation on ACD, with consideration of non-linear and delayed dependencies. The analysis uses an interesting approach of cubic splines and cross-basis functions in order to allow for nonlinearity and assessment of lags through relative risk with 95% CIs but the methods could be elaborated to allow the general reader (and even statisticians) to understand the approach a little better. The same goes for the meta-regression and the hypothesis tests being conducted. Overall, this is a nice extension to some work related to seasonality and diarrheal pathogens with a unique approach for showing nonlinearity and lag.

Response: Thank you sincerely for your kind words of appreciation. Based on your insightful feedback in the method, results, and conclusion sections, we have diligently incorporated your valuable comments and suggestions.

***Reviewer #2’s Comments***

***Method***

1. The methods used in this study are appropriate, comprehensive and well-articulated. I just have some questions:

Response: Thank you so much for the encouraging notes.

2. What was the rationale behind the selection of a 21-day rolling sum of daily total precipitation, as mentioned in lines 116-118. While the authors have appropriately cited three relevant references utilizing a similar lag period, it would be valuable to explicitly articulate the reasons for this choice and offer insights into the rationale for other lag times.

Response: Thank you so much for your kind suggestions. Firstly, we determined a 21-day period prior to the hospital visit as our exposure window. We assume that considering precipitation occurrences, transmission of pathogens, subsequent onset of diarrhoea infections, and hospital visits, these events are likely to unfold over approximately 21 days.. Although "rolling sum" may seem technical, they refer to the same concept regarding delayed effect. We have followed your suggestions and added the rationale to the manuscript line 133-137. Secondly, we also did sensitivity analysis with 14-day and 28-day rolling sums to justify using a 21-day rolling sum of precipitation results, and we found that our 21-day rolling sum of precipitation results was fairly robust for each site. Supplementary S7 Fig. (**Page 5, Line 139-143**).

3. The authors mention the utilization of a time series regression with a quasi-Poisson distribution. Could the authors elaborate on their approach to addressing overdispersion? Did they employ a robust sandwich estimator for standard errors? Additionally, if the authors used Generalized Additive Models for the spline fits, kindly specify this in the methods section.

Response: Thank you so much for your suggestions. Firstly, we have made a brief addition to the method section that explains how overdispersion is addressed. Secondly, we utilized a generalized linear model (GLM) with a quasi-Poisson function to address the assumption of no overdispersion instead of employing a robust sandwich estimator to overcome the violation of OLS assumptions such as homoscedasticity. Thirdly, Generalized Additive Models (GAM) and GLM play the same roles for the spline fit and cross-basis function. (**Page 5, Line 150-151**).

4. Combining WASH-related variables, specifically treated water and handwashing, to evaluate their combined impact on the association between temperature or precipitation and ACD might provide valuable insights. A study found that combined WASH had more reductions in diarrhea prevalence than water intervention alone (<https://linkinghub.elsevier.com/retrieve/pii/S2214109X17304904>).

Response: Thank you so much for your suggestions. Combined WASH indicators are not available for the site-specific setting. However, we have added the explanation of our non-significant association between the single WASH indicators and relative risk in the discussion section by citing the suggested [paper](https://linkinghub.elsevier.com/retrieve/pii/S2214109X17304904). (**Page 15, Line 346-351**).

5. Consideration of urbanicity/rurality of the study sites could be valuable. I suggest exploring the addition of these variables in the meta-regression or perhaps something to be discussed in the Discussion section.

Response: Many thanks for your valuable suggestions. We have added the "Urbanization" variable to our method in the meta-regression section. (**Page 6, Line 177-178, Table 3 and supplementary S3 Table**).

6. Further clarification is needed on the meta-analysis methodology. Specifically, could the authors confirm whether a two-stage hierarchical approach, as they referenced Sera and Gasparrini et al. 2022, was adopted? If so, please explicitly outline this in the methods section.

Response: Thank you so much for your kind suggestions. We have addressed your suggestions. (**Page 5-6, Line 149-150, 170-171**).

***Results***

7. The figures presented in the results section are visually appealing. Optional: The authors may try having the same scales and range for the x- and y-axes in Fig 1 and 2 for each study site for easier comparison.

Response: Thank you so much for your kind appreciations and suggestions. Based on suggestions, we have replaced Fig 1 and 2 to use consistent x- and y-axis. (**Page 11 and 12)**.

8. Regarding lines 177-178, where the authors suggest a linear association between temperature and ACD in most countries. Based on Fig 1 and Fig S1, it seems that the relationships are non-linear. Could the authors clarify?

Response: Many thanks for your concerns. We have corrected and clarified it. (**Page 8, Line 216**).

9. The non-significant findings of all wealth and economic variables in the meta-regression is a bit surprising since previous studies found association between socioeconomic position/status and child diarrhea. I'm wondering if the authors could explore more granular measures of wealth, such as an asset-based wealth index or maternal education in the analysis?

Response: Thank you so much for your suggestions. We collected records of all-cause diarrhea hospital visits (ACD) from the GEMS database registration for this study rather than moderate to severe diarrhoea (MSD). Although original GEMS (case-control) include individual information on demographic, socio-economic, epidemiological, clinical details, physical findings, and healthcare expenditures. Our ACD dataset does not include detailed information on wealth, such as an asset-based wealth index or maternal education. However, we agree with the reviewer that using such data may provide more information and help us detect any associations between the wealth/economic variables and the risk of temperature/precipitation on diarrhea.

10. Additionally, including descriptive statistics of the variables included in the meta-regression in Table 1, along with asset-based wealth index/maternal education and urbanicity/rurality, if applicable, would enhance the contextual understanding of each study site.

Response: Thank you once again. We have briefly described meta-predictors in the supplementary file (**S3 Table**).

11. Clarification is needed whenever the authors refer to "independent temperature/precipitation." Does this imply that these exposures were modeled separately or controlled for each other?

Response: Many thanks for your concerns. Yes, we have controlled temperature and precipitation for each other in the same model. (**Page 6, Line 163-164**).

***Conclusions***

12. Line 339 mentions "ambient temperature..." Could the authors clarify the term "ambient"? In my understanding, it could refer to indoor temperature, and this may affect how people might interpret the conclusion.

Response: Ambient temperature refers to the air temperature of the surrounding environment around an object or within a space. This value is also known as the outdoor temperature.

13. Also the authors acknowledged in their limitation that the study sites might not be nationally-representative. It might be beneficial to address this consideration in the conclusion when referring to the countries, for example, by specifying "certain areas/regions in Bangladesh."

Response: Thank you so much for your suggestions. According to your suggestion, we have included certain sites/regions in the conclusion. Please refer to the track changes in the conclusion section.

***Editorial and Data Presentation Modifications***

14. The authors may consider using "multivariable" meta-analysis/meta-regression instead of "multivariate" since they assessed only one outcome with multiple predictors.

Response: Thank you for your valuable suggestion. We have addressed it on **Line 38, 170** and **299**.

**Summary and General Comments**

15. I appreciate the opportunity to review this manuscript. The paper is very well-written with interesting findings.

Response: Thank you sincerely for your kind words of appreciation.

***Reviewer #3’s Comments***

***Method***

1. For clarity, the authors should state early in the methods section that the GEMS study used a case-control design. Though other publications have reported the design of the GEMS study, it is difficult to follow this manuscript without more details about the design. For example, there are no details about how controls were sampled and what the underlying target population was in each site. Further, to contextualize the study, more details are needed on whether the sites were primary urban or rural.

Response: Thank you so much for your kind suggestions. In the method section, we have added more detailed information on the GEMS case-control design. According to your suggestion, we have added site information in the context of urban or rural. (**Page 4-5, Line 106-116, 117-121**)

2. The methods should justify why the investigators considered a rolling 21-day sum for precipitation to be appropriate to capture any pathogen transport following weather events and the incubation period for the pathogens that primarily cause diarrhea in these settings. For temperature, the statistical analysis section alludes to the use of a 21-day lag, but this is not clearly justified either.

Response: Thank you so much for your kind suggestions. The response to this comment was the same as the response to the earlier **Comment 2** from **Reviewer 2**. (**Page 5, Line 139-143**).

3. There are insufficient details about the data sources for covariates such as GDP, poverty level, use of treated water, nutritional status, etc. Were all of these collected as part of the GEMS study? How were these measured? It is difficult to assess the risk of bias without further details.

Response: Thank you once again for your thoughtful concerns. We have included a new table in the supplementary to elucidate the definition of variables, data source and methodology behind variable creation (**S3 Table**). (**Page 6, Line 172-181**).

4. As noted below, it would be valuable to investigate relationships with heavy rainfall and extreme heat. It might also be helpful to investigate relationships within different seasons.

Response: Thank you so much for your suggestions. We estimated the relative risk for temperature at the 95^th^ percentile (High) compared to the 1^st^ percentile (low temperature). Similarly, we estimated the relative risk for precipitation at the 95^th^ percentile (heavy rainfall) compared to 1^st^ percentile precipitation (low rainfall). In our study, we employed a time series model to explore the relationship between child diarrhoea with temperature and precipitation. However, conducting season-specific site-wise time series modeling is challenging due to the limited statistical power resulting from our small sample size. (**Page 16, Line 387-390**).

***Results***

5. The site-specific precipitation results have such wide confidence intervals that it is difficult to say much about the slope of the relationship in most of the countries (Mali, Mozambique, Bangladesh, Pakistan). The results presentation and discussion section should be modified to account for this.

Response: We appreciate your feedback regarding the wide confidence intervals observed in our site-specific precipitation results. We have addressed your suggestions in the results and discussion section. (**Page 9, 14, Line 240-242, 317-319**)

***Conclusions***

6. The discussion should not draw conclusions about the impact of climate change on diarrhea (e.g., line 301) but rather should focus on the association between weather and diarrhea risk.

Response: Thank you for your valuable suggestion. We have modified it on **Line 353**.

7. The authors state that investigating pathogen-specific relationships with weather will be crucial, but they did not do so even though they appeared to have the data for it. Was the reason not to look at bacterial diarrhea, viral diarrhea, etc. because of the limited sample size?

Response: Thank you so much for your thoughtful concerns. Firstly, we agree entirely that it is challenging to build virus or bacteria specific time series model due to the limited pathogen-specific sample size data from GEMS. Secondly, The GEMS project enrolled MSD patients (pathogen-specific) with some selection criteria, such as 8-9 cases from each age stratum in a 14-day window period. Thus, the probability of daily sampling is not equal, and this dataset is not suitable for time series regression. Thirdly, the all-cause diarrhoea data we used in our study has no pathogen-specific information as it has not been tested in a lab.

8. The factors identified in the meta-regression (ORS use, nutritional status) are not clearly linked to weather. It is not clear if the authors hypothesize that weather changes these risk factors or if they are just strongly associated with diarrhea-related healthcare utilization.

Response: We employed meta-regression techniques to investigate the reasons behind the varying associations between temperature-diarrhea and precipitation-diarrhea across different regions. Site-specific indicators related to socioeconomic factors (such as GDP per capita, population density, poverty level, and urbanization) and public health-related factors (including access to treated water, source of drinking water, handwashing practices, nutritional status, and use of oral rehydration solution at home) may differ by region and subsequently the risk of temperature and precipitation on diarrhoea. We have tried to clarify in the discussion section. (**Page 14, Line 307-309**)

***Editorial and Data Presentation Modifications***

9. In the abstract, it is not clear if the RRs are for a one-unit increase in temperature or precipitation.

Response: Thank you for your valuable suggestion. We have followed your suggestions and added RRs for a one-unit increase in temperature/precipitation. (**Page 2, Line 39, 42**)

10. On line 302, regarding the point about young children being exposed to contaminated water and food in the home, it seems just as likely that older children are exposed to more pathogens because they have exposures both inside and outside the home.

Response: We appreciate your thoughtful comments and the fact that you know more about the situation. We agree that older children may indeed have increased opportunities for exposure to various pathogens due to their more active engagement with the indoor and outdoor environment. As we mentioned, very young children tend to spend a significant amount of time indoors with their mothers, who are often engaged in domestic activities that involve handling contaminated water and food sources. This close proximity to potential sources of contamination within the household environment could contribute significantly to their increased susceptibility to diarrheal diseases. Furthermore, the physiological differences between very young children and older children, such as their developing immune systems and behaviors such as mouthing objects, may also play a role in their susceptibility to diarrheal diseases. We have added an explanation in **Line 357-360, Page 15**.

**Summary and General Comments**

11. This manuscript presents associations between temperature and precipitation and diarrhea using data from the GEMS study. The results differ substantially between sites, with some sites showing increased risk of diarrhea with increasing temperature and some showing decreased risk. The strength of relationships in opposite directions in different sites is somewhat surprising. It seems that the authors could do more to use the rich GEMS dataset to understand the heterogeneity in these findings. For example, they did not fully leverage pathogen data to understand how temperature and precipitation are related to bacterial vs. viral diarrhea. They also did not investigate relationships with indicators for heavy rainfall or extreme heat. In addition, unlike prior studies, they did not investigate whether the precipitation findings differed when restricting rainfall following dry periods. Most prior studies have found that diarrhea risk is higher following heavy rainfall if the preceding period was dry (concentration dilution hypothesis). The authors seemed to have the data to investigate this but did not do so.

Response: Thank you so much for your valuable comments and suggestions.

i. Why not pathogen-specific data analysis, i.e., bacterial vs viral?

Response: The response to this comment was the same as the response to the earlier **Comment 7** in the **Conclusion**.

ii. Investigations on heavy rainfall and extreme heat

Response: The response to this comment was the same as the response to the earlier **Comment 4** in the **Method**.

iii. Investigations on rainfall and diarrhoea association by restricting rainfall following dry periods.

Response: We appreciate the insightful feedback provided by the reviewer. While our study primarily investigates the nonlinear and delayed association between rainfall and diarrhea using time series methods, we acknowledge the significance of considering the concentration dilution hypothesis, which suggests that diarrhea risk may be higher following heavy rain if the preceding period was dry. The previous two studies [Carlton et al. 2014](https://academic.oup.com/aje/article/179/3/344/104219?login=true) and [Alexander et al. 2018](https://journals.plos.org/plosmedicine/article?id=10.1371/journal.pmed.1002688) limited their analyses to specific seasons. However, if we continue with the same approach and examine diarrhoeal patterns during the wet season following the dry seasons, we will encounter a limited sample size.

Although we did not specifically investigate the differential impact of precipitation following dry periods, we believe our study provides valuable insights into the overall relationship between rainfall and diarrhea incidence. However, we acknowledge that further investigation is needed to fully understand the concentration dilution hypothesis.
