# Supplementary material for: Short-term associations of diarrhoeal diseases in children with temperature and precipitation in seven low- and middle-income countries from Sub-Saharan Africa and South Asia in the Global Enteric Multicenter Study
Source: PLoS Negl Trop Dis. 2024 Oct 15;18(10):e0011834. doi: 10.1371/journal.pntd.0011834 (PMC11510124; doi:10.1371/journal.pntd.0011834)
Supplement: Attachment — Submitted filename: Response_PNTD-D-23-01533.docx [file pntd.0011834.s012.docx]

**Reviewers’ Comments**

**(Manuscript ID:** PNTD-D-23-01533**)**

**Manuscript Title:** Short-term associations of diarrhoeal diseases in children with temperature and precipitation in seven low- and middle-income countries from Sub-Saharan Africa and South Asia in the Global Enteric Multicenter Study

Dear Editor,

Thank you so much for the opportunity to revise our manuscript once again, and we gratefully acknowledge the reviewers for their insightful comments that undoubtedly would improve the quality of our manuscript. We have now revised the manuscript accordingly. Our point-by-point response to the reviewers’ comments is enclosed below in blue font. The page number refers to the revised version with track changes.

***Reviewer #1’s Comments***

***Summary and General Comments***

1. Overall I think the revised manuscript looks great. The strengths and perhaps novelty of the study include the interesting dlnm approach for assessing associations between weather variables and diarrhea which includes an exploration of temperature effects over 21 lag days. The meta analysis section is quite brief in the paper and as a result, the results are slightly hard to follow and interpret. I think a little more in-depth explanation of the metaanalysis and regression could be included in methods for clarity.
1. Response: Thank you so much for reviewing our manuscript and providing your positive feedback. We appreciate your acknowledgment of our study's strengths and novelty. We understand your concern regarding the meta-analysis section and the clarity of the results. To address this, we provided a more detailed explanation of the meta-analysis in the methods section, and according to your suggestions, we have revised the results of Table 3. We hope the interpretation of the results enhanced clarity and facilitated understanding.

Thank you for your valuable input, which helped improve the manuscript.

***Methods***

2. See my comments in results. I think the critique could be mostly addressed in methods.

Response: Thank you so much for your kind suggestions.

***Results***

3. The meta-analysis results (Table 3) are still a little difficult to follow. Though the additional explanation of the hypothesis tests in methods was helpful, I think the authors could be very clear that the metaanalyis is done on the estimate (as relative risk?) for the association between Temp and Diarrhea or Prec. and Diarrhea and the predictors are accounting for additional variance. After understanding that, it's clear how you might additionally adjust for temp and precipitation but it's not stated in methods. It's also not clear if the metaregression/analysis is done on patient-level data or site-level data. Finally, would it be possible to include effect estimates of the predictors in addition to the I-squared value and p-values?

Response: Thank you so much for your valuable comments and suggestions.

i. It's clear how you might additionally adjust for temperature and precipitation, but this is not stated in the methods.

Response: We have included an explanation in the methods section. (**Page 7, Line 207-209**).

ii. It's also not clear if the meta-regression/analysis is done on patient-level data or site-level data.

Response: We have included site-level related indicators to conduct meta-regression. (**Page 7, Line 202**).

iii. Would it be possible to include effect estimates of the predictors in addition to the *I*-squared value and p-values?

Response: We followed your suggestions by adding the effect estimates to **Table 3**. The coefficient represents the association between each meta-predictor and the RR. RR is estimated as the risk ratio at the 95^th^ percentile compared to the risk at the 1^st^ percentile. (**Page 11, Line 284, Table 3**)

***Conclusions***

4. No new comments

***Editorial and Data Presentation Modifications***

5. No new comments

***Reviewer #2’s Comments***

***Summary and General Comments***

1. I would like to thank the authors for the changes made in the manuscript. I think this is a very interesting and timely paper. I only have a minor comment/ suggestion before the acceptance of the manuscript which is regarding lines 346-351 (of the draft with track changes). I would like to clarify that in the cited article (<https://linkinghub.elsevier.com/retrieve/pii/S2214109X17304904>), there was no significant reduction in diarrhea prevalence for single water intervention. But for single sanitation and handwashing interventions, they were significant reductions which were almost similar with the effects of combined WSH and combined WSH + nutrition in rural Bangladesh.

Response: We have modified the sentence. (**Page 16, Line 375-379**).

Thank you so much for reviewing our manuscript. We are pleased to hear that you found the paper interesting and timely. Your comments and suggestions were invaluable in enhancing our research work.

***Method***

2. The authors have answered my questions regarding the Methods and made the necessary changes based on my comments.

Response: Thank you so much for your kind appreciation words and encouraging notes.

***Results***

3. I have no further comments. Thank you to the authors for making the changes accordingly.

Response: Thank you so much.

***Conclusions***

4. I have no further comments.

***Editorial and Data Presentation Modifications***

5. (No Response)

***Reviewer #3’s Comments***

***Summary and General Comments***

1. Thank you for the opportunity to review this paper. This work addresses an important research gap and demonstrates how associations between weather and diarrhea varies across different settings. The authors successfully integrated data from GEMS and ERA-5, as well as various data products capturing socioeconomic factors, to complete a comprehensive assessment of factors that may influence these relationships. The inclusion of GDP, poverty, population density, and other health indicators was unique and provide a strong foundation for future studies that will investigate these relationships. However, there are some inconsistencies in how the methods and results were reported throughout the manuscript. These discrepancies should be resolved prior to publication, to allow for more clear interpretation of these important results.

Response: Thank you for your thorough and insightful review of our paper. We are pleased to hear that you recognize the importance of our work. We carefully reviewed and revised the manuscript to address these discrepancies, ensuring that our findings are presented in a clear and interpretable manner. Again, thank you for your constructive comments and the opportunity to improve our work.

***Method***

2. The authors use an appropriate data source for assessing meteorological variables, with the right time and space granularity needed for their analysis.

Response: Thank you so much for your kind appreciation words and encouraging notes.

3. Please state which temperature measurement (outdoor vs dew point) was used in the primary analysis.

Response: Thank you for your valuable suggestion. In this study, we used ambient temperature as primary exposure, also known as outdoor temperatures. **Line 145 and 172**.

4. Lines 133-134: “Although ‘rolling sum’ may seem technical, they refer to the same concept regarding delayed effect.”

- I don’t fully agree with this claim. While the rolling sum captures precipitation over a 21-day lookback period, it’s not the same as what’s happening with the distributed lags for temperature. For example, the 21-day lag temperature may describe the effect of temperature 21 days prior to the diarrhea event, independent of temperatures in subsequent days. The 21-day rolling sum of precipitation aggregates rainfall across the whole period, making it difficult to separate the impacts of precipitation within that time. In the most extreme case, the total precipitation might have been entirely concentrated on the same day as a diarrhea event and would not represent a delayed effect. If the authors would like to capture a delayed effect in precipitation, they could add a cross-basis function with lagged total precipitation (perhaps summed over a small window).

Response: Many thanks for your concerns. We acknowledge that the rolling sum of precipitation over 21 days and the distributed lags for temperature capture different aspects of the data. Specifically, the rolling sum aggregates rainfall over the entire period, which can obscure the specific timing of precipitation events. We have modified it on **Page 6, Lines 153-154**.

1. Lines 157-159: “The overall cumulative relative risk (RR) for both temperature and precipitation was estimated as the ratio of the risk at the 95^th^ percentile compared to the risk at the 1^st^ percentile of each variable.“ Based on Figures 1 and 2, it looks like cumulative RRs were also calculated for all other percentiles? This should be explicitly stated in the methods. Given that these are primary results, additional detail is also needed on how cumulative relative risks are estimated. It seems like they aggregate over all lagged temperatures, but how is this done? This information is needed to guide the interpretation of the results and should not solely rely on an external citation.

Response: Thank you so much for your valuable comments and suggestions. We have followed your suggestions and added more detailed explanations. (**Page 7, Line 186-190**)

***Results***

2. The abstract reports relative risks for diarrhea associated with 1 unit increases in temperature and precipitation. However, the reported results seem to correspond to the cumulative RRs that estimated differences in risk between the 1st vs 95th percentile values (as reported in Table 2). This statement also implies a strictly linear relationship between diarrhea and temperature + precipitation, but the authors have only assessed these relationships with non-linear models.

Response: Thank you so much for your comments. We have modified the abstract. (**Page 2 Line 39-40 and 42**)

In our study, we utilized non-linear models to assess the association between diarrhea and variations in temperature and precipitation. The exposure-response curves demonstrate various non-linear patterns. For instance, the temperature-diarrhea curve in India exhibited a non-linear shape. In Bangladesh, the risk increased between 17°C and 27°C and then decreased after 28°C, indicating a non-linear relationship. Other sites, such as Mali and Pakistan, also showed somewhat non-linear patterns. Although the associations in The Gambia and Mozambique appeared more linear, they were not strictly so. Similarly, most sites exhibited non-linear shapes for the precipitation-diarrhea association, with the exceptions of India and Kenya, where the relationships were more linear but still not strictly. We reported the relative risks (RRs) for extreme percentiles (1st vs. 95th) to capture the overall effect temperature and precipitation on diarrhea and to facilitate comparisons across different sites. However, it was challenging for us to build a model and present results in a consistent style across seven diverse sites.

5. Generally, cumulative RRs don’t have an intuitive interpretation for how temperature + precipitation impact diarrhea risk. The authors should be more specific in how they describe these estimates and avoid conflating it with the risk associated with a simple one-unit increase in temperature + precipitation.
i. It may be more clear to report and plot RRs for fixed lags and/or fixed temperature + precipitation values

Response: Thank you for your valuable comments and suggestions. The response to this comment was similar to the earlier **Comment 6** in the **Results**. Based on your suggestions, we have created fixed temperature and lag plots. Supplementary Figures **S3a-c**.

6. Throughout the results, the authors state how RR increases or decreases with weather but they seem to be describing trends in incidence.

I would recommend making the edits similar to below:

- Original [Lines 203-205]: The RR of diarrhoea increased in Mozambique (4.96, 95% confidence interval [CI] 2.81-8.76)
- Edited: The incidence of diarrhoea increased with temperature in Mozambique (RR [XX vs YY degrees C] = 4.96, 95% confidence interval [CI] 2.81-8.76)

Response: Thank you for your valuable suggestion. We have modified it on **Line 244-248, and 265-267**.

***Conclusions***

7. It would help to provide a brief description of the climate of each site, particularly in reference to the timing and severity of rainy seasons. This would also emphasize the climate diversity of sites selected for this study.

Response: Thank you once again for your suggestions. We have added line in the introduction section **Line 83-91 and 110-113**.

i. If the study only included diarrhea cases from hospitalized individuals, it would be important for authors to discuss how the exclusion of non-hospitalized diarrhea cases influences their results.

Response: Thank you once again for your thoughtful concerns. Our study included data from both hospitalized and non-hospitalized under-5 child diarrhea patients who visited the GEMS-nominated hospital. (**Page 4, Line 116-117**)

8. Lines 330-334: Previous studies have indicated that combined water, sanitation, and handwashing (WASH) interventions led to greater reductions in diarrhoea prevalence compared to single water, sanitation, or handwashing interventions. This might explain the weak evidence we observed for the association between relative risk with treated water, drinking water, and handwashing.

- A secondary analysis of this trial also found that the effect of combined WASH + sanitation interventions was concentrated in the monsoon season and following periods of heavy rainfall, but that water and handwashing interventions were less influenced by weather (https://ehp.niehs.nih.gov/doi/10.1289/EHP13807)

Response: Thank you so much for suggesting. We have cited this article in the discussion section. (**Page 16, Line 383-385**)

***Editorial and Data Presentation Modifications***

9. In the text of results, the authors describe trends shown in the figures to convey the direction of the weather-diarrhea relationships. (Lines 219-221: “The association between precipitation and ACD infection exhibited a monotonic increasing pattern … levelled off at higher precipitations… (Fig 2)”). However, it’s hard to see these trends in Figures 1 + 2 since all facets are plotted with the same y-axis scale. The authors should consider allowing the y-axis of the plots to vary by country or include more informative descriptions in the text.

Response: Thank you so much for your kind suggestions. Based on suggestions, we have replaced **Fig 1** and **Fig 2**, allowing the y-axis of the plots to vary by site.
